# Recurrent migraine with visual aura as the primary phenotype of familial neuronal intranuclear inclusion disease

**DOI:** 10.3389/fneur.2026.1747202

**Published:** 2026-02-11

**Authors:** Qingxiang Zhang, Min Gao, Yueshan Piao, Sufen Huang, Haitian Nan, Zhen Wang, Junjie Li

**Affiliations:** 1 Department of Neurology, Xuanwu Hospital, Capital Medical University, Beijing, China; 2 Department of Neurology, Dehua County Hospital, Quanzhou, China; 3 Department of Pathology, Xuanwu Hospital, Capital Medical University, Beijing, China; 4 Department of Neurology, Liuyang Jili Hospital, Changsha, China

**Keywords:** headache, migraine with aura, negative cranial MRI findings, neuronal intranuclear inclusion disease, *NOTCH2NLC*

## Abstract

Neuronal intranuclear inclusion disease (NIID) is a rare neurodegenerative disorder characterized by highly heterogeneous clinical manifestations and multi-system involvement. The most common initial symptoms include tremor, cognitive impairment, and muscle weakness. Characteristic neuroimaging features comprise symmetrical diffusion-weighted imaging (DWI) high signal intensity in the corticomedullary junction and extensive leukoencephalopathy. NIID manifesting as migraine with visual aura as the predominant symptom has rarely been reported. In this study, we describe a Chinese NIID pedigree comprising eight affected members, all of whom consistently exhibited migraine with visual aura as the primary clinical feature. Notably, none of the followed-up patients showed abnormalities on neuroimaging. In one case, serial follow-up over 7 years revealed no abnormal DWI high signal intensity at the corticomedullary junction or leukoencephalopathy. Skin biopsies confirmed the presence of neuronal intranuclear inclusions in two affected patients within this pedigree. Genetic testing for the NIID-causing mutation identified the GGC repeat expansion in the *NOTCH2NLC* gene in three patients in this family. This study provides new insights into the phenotypic complexity of NIID.

## 1 Introduction

Neuronal intranuclear inclusion disease (NIID) is a rare neurodegenerative disorder characterized by highly heterogeneous clinical manifestations and multi-system involvement (1, 2). The clinical spectrum encompasses cognitive dysfunction (3), peripheral neuropathy (4, 5), movement disorders (6–8), paroxysmal symptoms (9, 10), and autonomic dysfunction (4). The most common initial symptoms include tremor, cognitive impairment (2, 11, 12), and muscle weakness (13). Patients with a paroxysmal presentation experience acute or recurrent episodic events, including impaired consciousness, stroke-like episodes, encephalitis-like attacks, and seizures (14). NIID predominantly manifesting as recurrent migraine with visual aura has rarely been reported. In this study we report the first pedigree of NIID manifesting as migraine with aura as the major symptom. All affected members within the pedigree presented with migraine with visual aura as their primary clinical manifestation. Notably, none of the followed-up patients exhibited abnormalities on neuroimaging. In one case, serial follow-up over 7 years revealed no abnormal DWI high signal intensity at the corticomedullary junction or evidence of leukoencephalopathy.

## 2 Methods

### 2.1 DNA isolation and the whole-exome sequencing (WES) study

Genomic DNA was extracted from peripheral blood lymphocytes following a standard protocol. All DNA samples were normalized to 50–100 ng/μl. WES was performed on the patient’s genomic DNA. We summarized migraine, stroke-like episodes, leukodystrophy, and dementia -related and susceptible genes using the Online Mendelian Inheritance in Man (OMIM) and PubMed database. Exome capture was performed with a SureSelect Human All Exon V6 + UTR (89 Mb) Kit (Agilent Technologies, Santa Clara, CA, USA). Paired-end sequencing was carried out on a HiSeq2500 (Illumina, San Diego, CA, USA) using a HiSeq SBS Kit V4 (Illumina), which generated 100-bp reads. The average and minimum sequencing depths were 205 × and 10×, respectively. The reference databases utilized included GRCh38/hg38,^1^ HGMD,^2^ ExAC,^3^ 1,000 Genome,^4^ gnomAD,^5^ ClinVar,^6^ and dbSNP.^7^ WES data were analyzed for single-nucleotide variants (SNVs) and insertion-deletions (InDels) in dementia-related causing and susceptible genes. Genetic variants were classified as “predicted deleterious” through consensus predictions from five established bioinformatics tools: PolyPhen-2^8^ with scores >0.95 (considered “probably damaging”), SIFT^9^ with scores ≤0.05 (deleterious threshold), PROVEAN (see Footnote 10) with scores ≤ −2.5 (deleterious cutoff), MutationTaster^10^ using default parameters (probability >0.99), CADD^11^ with phred-scaled scores >20 (top 1% of deleterious variants). The significant results were comprehensively evaluated in aspects including minor allele frequency, conservation, predicted pathogenicity, disease association, and confirmation with Sanger sequencing. All heterozygous variants with a minor allele frequency < 0.1%, as well as homozygous and potentially compound heterozygous variants, were considered. Variants were classified as pathogenic or likely pathogenic based on the guidelines of the American College of Medical Genetics and Genomics (ACMG). Copy number variation (CNV) calling was performed using ExomeDepth. Variant filtering focused on a predefined panel of genes associated with familial hemiplegic migraine (e.g., *CACNA1A, ATP1A2, SCN1A*), cerebral autosomal dominant arteriopathy (e.g., *NOTCH3*), and other hereditary neurodegenerative disorders. Mitochondrial gene mutation analysis was performed via next-generation sequencing of the entire mitochondrial genome to rule out mitochondrial encephalomyopathy, lactic acidosis, and stroke-like episodes (MELAS), with a heteroplasmy detection threshold of >5%.

### 2.2 Skin biopsy and immunohistochemistry

Skin punch biopsies (3 mm diameter) were obtained from the distal leg, approximately 10 cm above the lateral malleolus, which is the standard site for NIID diagnosis. Tissue samples were fixed in 10% neutral buffered formalin and embedded in paraffin. Serial sections (4 μm) were stained with hematoxylin and eosin. Immunohistochemical staining was performed using anti-p62 (Abcam, ab56416, 1:200 dilution) and anti-ubiquitin (Dako, 1:100 dilution) antibodies. The presence of intranuclear inclusions was assessed in adipocytes, fibroblasts, and sweat gland duct epithelial cells.

### 2.3 *NOTCH2NLC* repeat expansion screening

Screening for GGC repeat expansions in the 5′ UTR of the *NOTCH2NLC* gene was performed using Repeat-Primed PCR (RP-PCR). The primer sequences used were: Forward: 5′-FAM-GGCATTTGCGCCTGTGCTTCGGACCGT-3′; Reverse (M13-linker): 5′-CAGGAAACAGCTATGACC-3′; and Reverse (repeat-targeting): 5′-CAGGAAACAGCTATGACCTCCTCCGCCGCCGCCGCC-3′. Fragment analysis was performed on an ABI 3730xl Genetic Analyzer (Applied Biosystems) to identify the characteristic sawtooth pattern. The precise repeat number was determined using fluorescence amplicon length PCR (AL-PCR). A repeat size of >60 GGC repeats was considered pathogenic.

## 3 Case presentation

**Figure 1:**
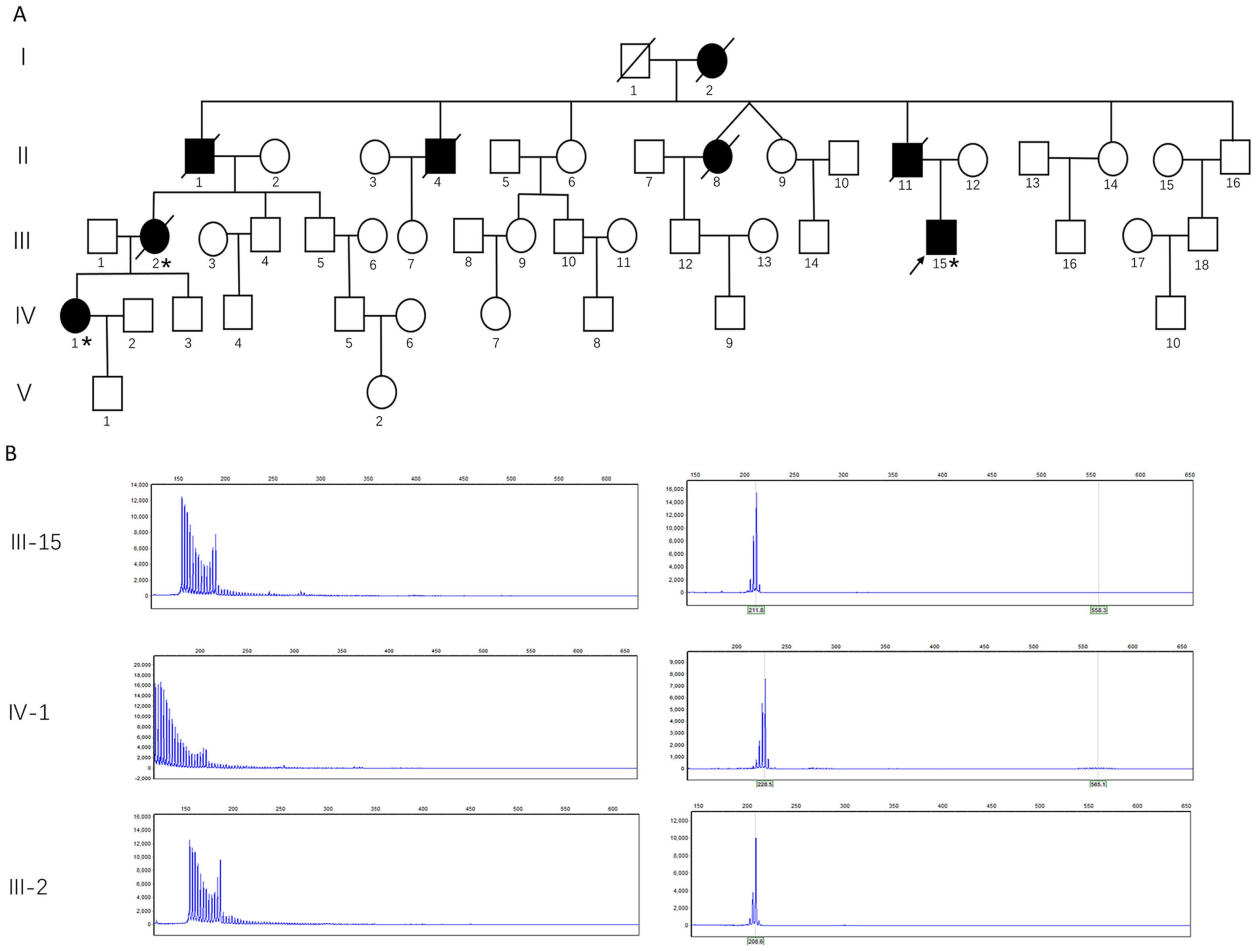
Pedigree tree and the genetic study of this family. **(A)** Pedigree tree of the family with the repeat expansion mutation in *NOTCH2NLC*. There were eight symptomatic patients in five generations of this pedigree. The proband is indicated (arrow). Squares indicate men, circles women, and slashes deceased individuals, while shaded (black) symbols indicate individuals with symptoms of NIID, while unshaded ones show individuals without symptoms of NIID. Individuals evaluated clinically or genetically are denoted by asterisks. **(B)** The genetic study of the three patients in this pedigree. Repeat-primed PCR combined with high-resolution pulsed-field capillary electrophoresis analysis revealed that the proband (III-15), the proband’s niece (IV-1), and the proband’s sister (III-2) carry the *NOTCH2NLC* repeat expansion. Results of repeat primed PCR for *NOTCH2NLC* expansion demonstrating the saw-tooth pattern (left panel), typical of the pathological expansion. Using high-resolution pulsed-field capillary electrophoresis analysis (right panel), GGC trinucleotide repeat expansions were measured at 135, 137, and 104 repeats in the proband (III-15), the proband’s niece (IV-1), and the proband’s sister (III-2), respectively.

**Figure 2:**
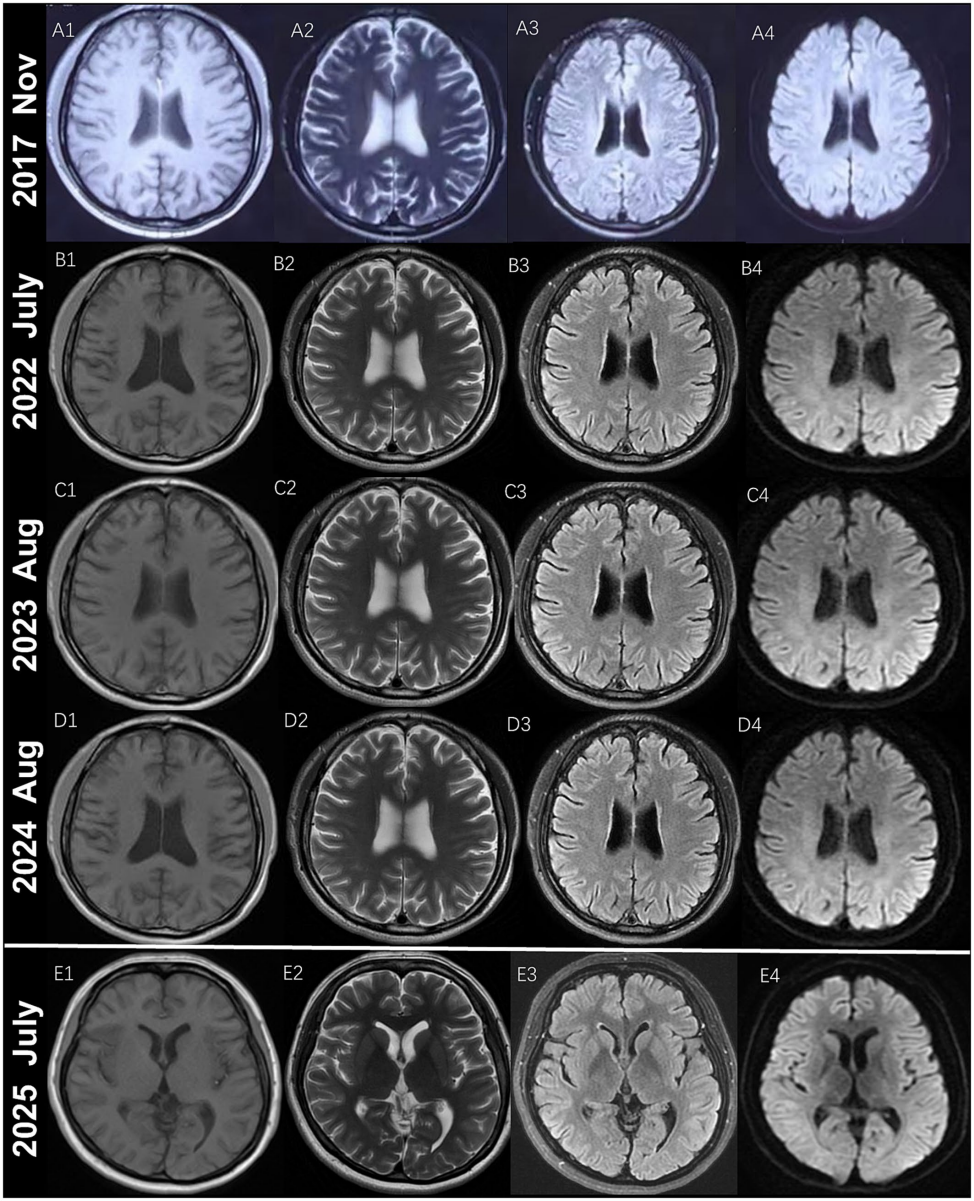
Neuroimaging findings in two NIID patients from the pedigree. Cranial MRI of Patient 1 (Figure 1A, III-15) and Patient 2 (Figure 1A, IV-1). Scans **(A–D)** correspond to Patient 1, while scan **(E)** represents Patient 2. Representative axial images include: T1-weighted imaging **(A1,B1,C1,D1,E1)**, T2-weighted imaging **(A2,B2,C2,D2,E2)**, diffusion-weighted imaging (DWI) **(A3,B3,C3,D3,E3)**, and T2 fluid-attenuated inversion recovery (FLAIR) sequences **(A4,B4,C4,D4,E4)**. Scan timepoints: **(A)** 2017 **(A1–A4)**; **(B)** 2022 **(B1–B4)**; **(C)** 2023 **(C1–C4)**; **(D)** 2024 **(D1–D4)**; **(E)** 2025 **(E1–E4)**.

**Figure 3:**
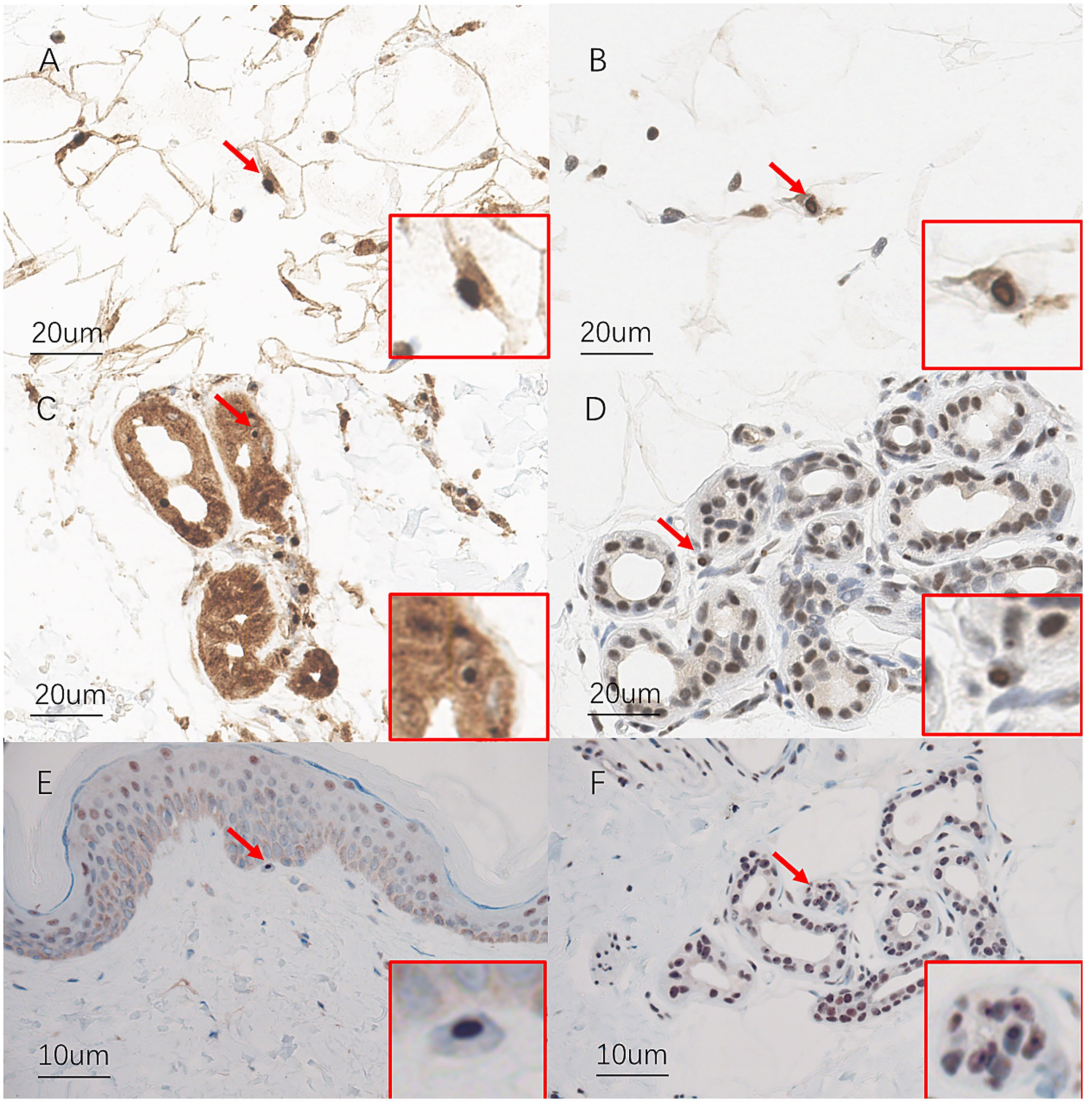
Pathological study of the patients. Pathological findings in case 1 **(A–D)** and case 2 **(E,F)**. **(A)** P62-positive intranuclear inclusions in adipocytes. **(B)** Ubiquitin-positive intranuclear inclusions in adipocytes. **(C)** P62-positive intranuclear inclusions in sebaceous gland cells. **(D)** Ubiquitin-positive intranuclear inclusions in sebaceous gland cells. **(E)** P62-positive intranuclear inclusions in dermal cells. **(F)** Ubiquitin-positive intranuclear inclusions in eccrine gland cells.

Case 1 (Figure 1A, III-15): A 35-year-old male patient developed migraine with visual aura at the age of 30. During the aura phase, he reported a central bright spot in the binocular visual field, which gradually expanded and increased in size, accompanied by a water-ripple-like sensation and photopsia. This was associated with blurred vision and decreased visual acuity, lasting 30–40 min before resolution. Approximately 30 to 60 min later, the patient developed a fever of 38 °C–39 °C, accompanied by headache and chills. The headache was predominantly characterized by a bilateral, distending pain across the frontal, temporal, and occipital regions. Occasionally, it manifested as a throbbing or stabbing sensation, with an intensity rated 7–8 out of 10. It was associated with explosive ocular pain, nausea, photophobia, and phonophobia, but no vomiting. During attacks, the patient exhibited lethargy, irritability, and reduced speech. Symptoms usually completely resolved within 3–5 h after taking ibuprofen. Initially, the headaches occurred 1–2 times per year. At that time, cranial magnetic resonance imaging (MRI) revealed no significant abnormalities (Figure 2A), while electroencephalography (EEG) showed an increase in generalized theta wave activity. Two years after onset, following a 30–40 min visual aura, the patient developed non-fixed limb numbness and weakness spreading from distal to proximal regions, accompanied by dysarthria. These symptoms persisted for 40–60 min. One to two hours later, a headache resembling prior episodes developed. In the same year, interictal visual disturbances emerged, including metamorphopsia and chromatic aberration in the right eye. Four years after onset, the headache attacks gradually increased in frequency, reaching up to 2–3 episodes per month. Repeat cranial MRI showed no significant abnormalities (Figure 2B). Five years after initial presentation, the patient developed interictal visual acuity decline in the right eye and memory impairment. Upon admission, physical examination revealed bilateral visual acuity decline, with right eye vision limited to counting fingers at 1 foot and left eye acuity of 0.4. The patient exhibited impaired recent memory, while remote memory, calculation ability, and orientation remained intact. Tendon reflexes were diminished in all four limbs. Cognitive assessment scores were 30 on the Mini-Mental State Examination (MMSE) and 26 on the Montreal Cognitive Assessment (MoCA). No significant abnormalities were noted in past medical history. The third cranial MRI demonstrated no significant abnormalities (Figure 2C). Lumbar puncture revealed unremarkable cerebrospinal fluid findings. Funduscopic examination identified bilateral macular edema. EEG demonstrated an increase in generalized theta wave activity. Whole-exome sequencing and mitochondrial gene mutation analysis revealed no definitive pathogenic variants associated with migraine with aura or other neurological disorders. Treatment with topiramate was initiated, resulting in a reduction of headache frequency to 1–2 episodes per month. Six years after onset, the patient returned to our hospital for follow-up due to significantly increased headache frequency and further visual deterioration. Repeat cranial MRI again showed no significant abnormalities (Figure 2D). Electrophysiological studies revealed a pattern of peripheral neuropathy predominantly characterized by demyelination, with reduced motor and sensory nerve conduction velocities in all four limbs and preserved amplitudes. Skin biopsy revealed ubiquitin- and p62-positive intranuclear inclusions in the eccrine sweat gland duct epithelium and adipocytes on immunohistochemical analysis (Figures 3A–D). Among the patient’s family members, a total of eight individuals were found to have similar symptoms (Figure 1A). Analysis of CGG repeat expansions in the 5′ untranslated region (5′ UTR) of the *NOTCH2NLC* gene using PCR-capillary electrophoresis and RP-PCR revealed GGC repeat numbers of 135 and 19 in the patient, supporting the diagnosis of NIID (Figure 1B).

Case 2 (Figure 1A, IV-1): The proband’s niece. A 30-year-old female patient began experiencing migraine with visual aura 3 years ago. The aura began with approximately 10 min of monocular photopsia and blurred vision. This was followed by a 30-min episode of numbness that progressed from distal to proximal in all four limbs, perioral numbness, limb clumsiness, and dysarthria. Subsequently, a tightening-type headache developed, localized primarily to the right parieto-occipital or bilateral temporal regions. The headache intensity reached a maximum score of 10/10 and was accompanied by photophobia, phonophobia, nausea, and vomiting. The patient experienced occasional low-grade fever. Headache episodes typically lasted 5–6 h, with prolonged episodes exceeding 10 h. The headache was alleviated by taking analgesics during attacks. Multiple doses of pain medication were sometimes required to manage a single headache episode. Initially, the headaches occurred 1–2 times per year. Six months ago, the frequency of headache attacks increased to once per month. During the disease course, the patient also experienced dry cough (with sensations of airway spasms and suffocation in severe cases), decreased appetite, and frequent loose stools. The patient experienced significant interictal visual decline, along with self-reported memory impairment over the three-year course of the disease. EEG revealed an increase in generalized theta wave activity. On admission, mild memory impairment was noted, while calculation ability and orientation remained intact. Bilateral visual acuity was reduced to 0.1 in the right eye and 0.4 in the left eye. Muscle tone was decreased in all four limbs, and tendon reflexes were diminished. Cognitive assessment scores were 28 on the MMSE and 24 on the MoCA. Non-contrast and contrast-enhanced cranial MRI revealed no definite abnormalities (Figure 2E). Nerve conduction studies demonstrated a pattern of peripheral neuropathy predominantly characterized by demyelination, with reduced motor and sensory nerve conduction velocities in all four limbs and preserved amplitudes. Fundus photography demonstrated bilateral macular edema. Skin biopsy revealed scattered ubiquitin- and p62-positive intranuclear inclusions within eccrine gland cells (Figures 3E,F). Whole-exome and mitochondrial genetic testing revealed no definitive pathogenic variants associated with the clinical phenotype. GGC repeat expansion analysis identified GGC trinucleotide repeat expansions in the NIID-associated *NOTCH2NLC* gene, with repeat numbers of 137 and 25, respectively (Figure 1B).

Case 3 (Figure 1A, III-2): The proband’s paternal cousin and the mother of Case 2. The patient first developed migraine with visual aura at the age of 35. The aura phase manifested as monocular photopsia, which persisted for approximately 30 min before resolution. This was followed by an explosive-type headache predominantly localized to the right temporal region, with a pain intensity score of 10/10 accompanied by nausea and vomiting. Headache episodes lasted from several hours to up to 3 days, frequently occurring during menstruation and seasonal transitions. The frequency of attacks significantly decreased after menopause. The patient began experiencing visual decline and memory impairment at age 48. Unintentional weight loss developed at age 50. According to family members, the patient had sought medical attention at multiple hospitals. Cranial MRI examinations performed repeatedly between ages 37 and 53 showed no significant abnormalities. At age 53, RP-PCR and capillary electrophoresis analysis revealed GGC trinucleotide repeat expansions in the NIID-associated *NOTCH2NLC* gene, with repeat numbers of 104 and 18, respectively (Figure 1B). A final migraine episode occurred when the patient was 54 years old, presenting with a familiar visual aura. Both the patient and family, believing it to be a typical attack, initiated management with oral analgesics and bed rest. The patient was discovered deceased several hours later. The suspected cause is sudden cardiorespiratory arrest that occurred during the migraine.

Five additional family members were affected by a similar condition and are now deceased. These individuals comprised the proband’s grandmother (I-2), father (II-11), two paternal uncles (II-1, II-4), and one paternal aunt (II-8) (Figure 1A).

The proband’s grandmother (Figure 1A, I-2): Symptom onset occurred at age 60. According to family reports, her clinical presentation was characterized by migraine with visual aura as the initial and predominant symptom. The disease course was accompanied by cognitive decline and visual deterioration. She passed away in her early sixties.

The proband’s father (Figure 1A, II-11): Developed migraine with visual aura at age 35. He subsequently exhibited lethargy and drowsiness during attacks, along with progressive bilateral visual decline during interictal periods. At age 46, he experienced an 8-day episode of impaired consciousness following a migraine attack, with suspected epileptic seizures during this period. Mechanical ventilation via endotracheal intubation was required for 3 days, after which his consciousness cleared. In the same year, significant visual and memory deterioration began. By age 48, he had complete bilateral vision loss and became progressively bedbound. Weight loss and anorexia started at age 49, followed by gradual progression to mutism and decreased mobility. He passed away at age 56.

The proband’s paternal uncle (Figure 1A, II-1): Developed migraine with visual aura at age 48. The aura phase manifested as monocular photopsia accompanied by blurred vision, followed by severe headache with nausea and vomiting. Headache episodes lasted from several hours to multiple days. Cognitive decline emerged in his early fifties. He passed away at age 58.

The proband’s paternal uncle (Figure 1A, II-4): Developed symptoms in his early forties. According to family descriptions, he initially presented with unilateral cephalgia preceded by indescribable photic aura. Cognitive decline emerged in the later stages of the disease. He passed away at age 58.

The proband’s paternal aunt (Figure 1A, II-8): Developed symptoms in her early forties. According to family reports, her clinical presentation was characterized by migraine with visual aura as the initial and predominant symptom. The disease course was accompanied by cognitive decline and visual deterioration. She passed away at age 52. The clinical characteristics of the eight affected family members are summarized in Table 1.

**Table 1:** Clinical characteristics of the eight affected family members.

**ID**	**Sex**	**Age at onset**	**Core phenotype**	**MRI status (age at examination)**	**Skin biopsy**	***NOTCH2NLC* repeat length**	**Outcome (age at death)/suspected cause**
Patient I-2	Female	60	Migraine with visual aura, cognitive decline, and visual deterioration.	NA	NA	NA	Deceased (60+)
Patient II-1	Male	48	Migraine with visual aura, cognitive decline emerged in his early fifties.	NA	NA	NA	Deceased (58)
Patient II-4	Male	40+	Migraine with visual aura, cognitive decline emerged in the later stages of the disease.	NA	NA	NA	Deceased (58)
Patient II-8	Female	40+	Migraine with visual aura, cognitive decline, and visual deterioration.	NA	NA	NA	Deceased (52)
Patient II-11	Male	35	Migraine with visual aura, cognitive decline, and visual impairment. After migraine: 8-day impaired consciousness with suspected seizures, requiring 3-day ventilation. Progressed to bilateral blindness (age 48), bedbound state, then weight loss/anorexia (age 49), mutism, and immobility.	NA	NA	NA	Deceased (56)
Patient III-2	Female	35	Migraine with visual aura, experiencing visual decline and memory impairment at age 48. Unintentional weight loss developed at age 50.	Unremarkable (37-53)	NA	104 and 18	Deceased (54)/ sudden cardiorespiratory arrest
Patient III-15	Male	30	Migraine with visual aura, with blurred vision, reduced visual acuity, fever, and memory impairment.	Unremarkable (30-37)	ubiquitin- and p62-positive intranuclear inclusions	135 and 19	Ambulatory
Patient IV-1	Female	27	Migraine with visual aura, visual deterioration, and memory decline. Dry cough and reduced appetite, gastrointestinal symptoms of increased frequency of loose stools, as well as neurosensory complaints.	Unremarkable (30)	ubiquitin- and p62-positive intranuclear inclusions	137 and 25	Ambulatory

MRI, magnetic resonance imaging; NA, not available.

## 4 Discussion

In this study, we report a five-generation pedigree comprising 30 family members, including 3 genetically confirmed NIID patients and 8 individuals exhibiting clinical manifestations of recurrent episodic symptoms. Among the affected members, 4 were male and 4 were female. All affected patients presented with migraine with visual aura as the primary clinical manifestation. Notably, one individual suffered sudden unexpected death during a migraine attack. Other accompanying symptoms included bilateral visual decline, macular edema, and memory impairment. The age of onset among family members ranged from 30 to 40 years, while the age of death ranged from 50 to 60 years. NIID was suspected in this pedigree due to the familial clustering of recurrent migraine-like episodes with visual aura, accompanied by progressive peripheral neuropathy, cognitive changes, and potential autonomic features, despite consistently normal neuroimaging findings. Notably, the symptoms experienced by Case 1 during headache attacks—including high-grade fever (up to 39 °C), profound lethargy or altered consciousness, as well as dysarthria and limb numbness/weakness lasting 40–60 min—are not characteristic of primary migraine. Instead, they may represent clinically significant paroxysmal phenomena associated with NIID.

Patients with NIID may exhibit a broad spectrum of clinical manifestations. The most common initial symptoms typically include cognitive impairment (2, 11, 12), tremor, and muscle weakness (13). Approximately 28.7% of patients present with episodic symptoms as the initial manifestation of the disease. Among those with predominantly episodic symptoms, over 85% are sporadic cases (14). Cases presenting with headache as the initial symptom are relatively uncommon in NIID, accounting for only 6.7% of patients (15). To our knowledge, no NIID pedigrees have been reported in which the clinical presentation is predominantly characterized by migraine with visual aura with unremarkable findings on serial MRI follow-up. Some patients initially present with recurrent migraine with aura and later develop acute encephalopathy-like episodes. Upon clinical evaluation, brain MRI revealed mild DWI hyperintensity at the corticomedullary junction, significant right cerebral edema, and cortical enhancement (16). In another case with clinical manifestations of migraine attacks, follow-up MRI performed 3 years after symptom onset revealed hyperintensity at the corticomedullary junction (17). NIID pedigrees have been reported with patients exhibiting non-specific headache and acute stroke-like episodes (18), as well as recurrent headache with limb numbness and weakness (19). However, all these cases developed the characteristic high-signal “ribbon sign” in the corticomedullary junction on DWI. We report for the first time a NIID pedigree characterized by a homogeneous clinical presentation of migraine with visual aura and unremarkable neuroimaging findings. The absence of typical MRI findings, even over a longitudinal follow-up extending to seven years, does not exclude a diagnosis of NIID. This underscores the diagnostic limitation of neuroimaging and emphasizes the necessity of integrating comprehensive clinical and pathological assessments.

The most characteristic neuroimaging feature in NIID patients is high signal intensity in the subcortical U-fibers on DWI (2, 12), which serves as the strongest and most accessible evidence to guide further diagnostic procedures such as skin biopsy (2, 20). Few studies have also reported NIID cases without DWI high signal intensity, though such presentations remain relatively uncommon (6, 21, 22). Another characteristic neuroimaging feature of NIID is leukoencephalopathy, which is observed in 82.3% of patients (23). Some studies suggest that leukoencephalopathy is the most common imaging finding in patients with paroxysmal symptoms (13). In early stages, involvement may be confined to the corpus callosum (24), middle cerebellar peduncles, and cerebellar vermis (25). Among patients with paroxysmal symptoms, 87.5–94.9% exhibit the classic linear high signal intensity on DWI, and 84.7% demonstrate severe white matter hyperintensity (11, 13). Additionally, approximately one-third of these patients may develop focal cortical abnormalities predominantly in the temporo-parieto-occipital regions, including cortical edema, enhancement, or marked focal cortical atrophy (15). In this pedigree, Case 1 showed no evidence of high signal intensity in the subcortical U-fibers on DWI or leukoencephalopathy despite undergoing dynamic brain MRI follow-up for up to 7 years. Similarly, Case 2 exhibited no abnormalities on MRI even 3 years after symptom onset. These findings suggest that NIID patients presenting with episodic migraine may maintain normal neuroimaging findings over an extended period.

Ocular involvement is an emerging phenotype of *NOTCH2NLC*-related disorders. Both Case 1 and Case 2 exhibited bilateral macular edema and significant visual acuity decline, which was documented by fundus photography and Optical Coherence Tomography (OCT). Alternative causes including diabetic retinopathy and venous occlusion were excluded. Recent studies have demonstrated that *NOTCH2NLC* repeat expansions can cause widespread retinal degeneration, with intranuclear inclusions present in the retinal ganglion cells and photoreceptors (26). The macular edema observed in our patients likely reflects this underlying NIID-associated retinopathy. This finding highlights the importance of comprehensive ophthalmologic evaluation, including fundus photography and OCT, in patients with suspected NIID, as visual symptoms may serve as an early biomarker.

The clinical heterogeneity of NIID may correlate with the length of GGC trinucleotide repeats in the *NOTCH2NLC* gene. Typically, GGC repeats exceeding 200 expansions are associated with a phenotype predominantly characterized by muscle weakness (6), Repeats ranging from 100 to 200 expansions tend to result in a dementia-predominant phenotype (6, 27), while repeats fewer than 100 expansions are linked to a Parkinson’s disease-predominant phenotype (8). The age of onset is negatively correlated with the length of GGC trinucleotide repeats in the *NOTCH2NLC* gene (12). For every 10-unit increase in GGC repeat length, the symptom onset occurs approximately one year earlier (13). In the pedigree we report, we observed that the parental generation of Case 1 developed symptoms at ages 48, early 40s, and 35, respectively. Case 1 manifested the disease at age 28, while Case 3 developed symptoms at age 37. Case 2, a direct descendant of Case 3, presented with symptoms at age 29. These observations are consistent with a preliminary pattern of genetic anticipation. Although previous studies have suggested that genetic anticipation may not exist (28), other reports have documented this phenomenon within individual pedigrees (13, 29). In this pedigree, Case 2 harbored 137 GGC trinucleotide repeats in the *NOTCH2NLC* gene, while Case 3 carried 104 repeats. This suggests that the observed genetic anticipation may be associated with an increase in GGC repeat length within a certain range (29). Furthermore, our findings indicate that GGC trinucleotide repeat expansions ranging from 100 to 130 manifest a clinical phenotype predominantly characterized by migraine with aura, suggesting a plausible genotype–phenotype correlation. However, as exact GGC repeat lengths were only available for three individuals, a definitive correlation between repeat expansion size and intergenerational anticipation cannot be statistically established in this cohort. Future studies with larger cohorts are required to validate these preliminary genotype–phenotype observations.

This study has several limitations: (1) onset ages in earlier generations rely on family recall, possibly introducing bias; (2) *NOTCH2NLC* repeat sizes were obtained only for three living patients; (3) autonomic symptoms were documented clinically but not systematically assessed with formal testing; (4) the small number of genetically confirmed cases precludes definitive conclusions about genotype–phenotype correlations.

## 5 Conclusion

In this study, we report a NIID pedigree in which all affected members consistently presented with recurrent migraine with visual aura. Our study expands the phenotypic spectrum of NIID and suggests a potential genotype–phenotype correlation. NIID should be considered in the differential diagnosis for patients with a family history who present predominantly with migraine with visual aura, even in the absence of cranial MRI findings such as DWI high-signal intensity or leukoencephalopathy over many years.

## Data Availability

The original contributions presented in the study are included in the article/supplementary material, further inquiries can be directed to the corresponding author.

## Footnotes

1
http://genome.ucsc.edu

2
https://portal.biobase-international.com

3
https://exac.broadinstitute.org/

4
https://www.internationalgenome.org/

5
http://gnomad.broadinstitute.org

6
https://www.ncbi.nlm.nih.gov/clinvar/

7
https://www.ncbi.nlm.nih.gov/SNP

8
http://genetics.bwh.harvard.edu/pph2/

9
http://provean.jcvi.org/genome_submit_2.php

10
http://www.mutationtaster.org

11
https://cadd.gs.washington.edu/score

## References

[1] Takahashi-Fujigasaki J. Neuronal intranuclear hyaline inclusion disease. Neuropathology. (2003) 23:351–9. doi: 10.1046/j.1440-1789.2003.00524.x, 14719553

[2] Sone J Mori K Inagaki T Katsumata R Takagi S Yokoi S . Clinicopathological features of adult-onset neuronal intranuclear inclusion disease. Brain. (2016) 139:3170–86. doi: 10.1093/brain/aww249, 27797808 PMC5382941

[3] Araki K Sone J Fujioka Y Masuda M Ohdake R Tanaka Y . Memory loss and frontal cognitive dysfunction in a patient with adult-onset neuronal intranuclear inclusion disease. Intern Med. (2016) 55:2281–4. doi: 10.2169/internalmedicine.55.5544, 27523009

[4] Sone J Hishikawa N Koike H Hattori N Hirayama M Nagamatsu M . Neuronal intranuclear hyaline inclusion disease showing motor-sensory and autonomic neuropathy. Neurology. (2005) 65:1538–43. doi: 10.1212/01.wnl.0000184490.22527.90, 16301479

[5] Liao YC Chang FP Huang HW Chen TB Chou YT Hsu SL . GGC repeat expansion of NOTCH2NLC in Taiwanese patients with inherited neuropathies. Neurology. (2022) 98:e199–206. doi: 10.1212/WNL.0000000000013008, 34675106

[6] Tian Y Wang JL Huang W Zeng S Jiao B Liu Z . Expansion of human-specific GGC repeat in neuronal Intranuclear inclusion disease-related disorders. Am J Hum Genet. (2019) 105:166–76. doi: 10.1016/j.ajhg.2019.05.013, 31178126 PMC6612530

[7] Sun QY Xu Q Tian Y Hu ZM Qin LX Yang JX . Expansion of GGC repeat in the human-specific NOTCH2NLC gene is associated with essential tremor. Brain. (2020) 143:222–33. doi: 10.1093/brain/awz372, 31819945

[8] Ma D Tan YJ Ng A Ng ASL Ong HL Sim W . Association of NOTCH2NLC repeat expansions with Parkinson disease. JAMA Neurol. (2020) 77:1559–63. doi: 10.1001/jamaneurol.2020.3023, 32852534 PMC7445625

[9] Fujita K Osaki Y Miyamoto R Shimatani Y Abe T Sumikura H . Neurologic attack and dynamic perfusion abnormality in neuronal intranuclear inclusion disease. Neurol Clin Pract. (2017) 7:e39–42. doi: 10.1212/CPJ.0000000000000389, 29431160 PMC5800705

[10] Lin P Jin H Yi KC He X-S Lin S-F Wu G . A case report of sporadic adult neuronal intranuclear inclusion disease (NIID) with stroke-like onset. Front Neurol. (2020) 11:530. doi: 10.3389/fneur.2020.00530, 32587570 PMC7298109

[11] Tian Y Zhou L Gao J Jiao B Zhang S Xiao Q . Clinical features of NOTCH2NLC-related neuronal intranuclear inclusion disease. J Neurol Neurosurg Psychiatry. (2022) 93:1289–98. doi: 10.1136/jnnp-2022-329772, 36150844 PMC9685690

[12] Zhu R Qu J Xu G Wu Y Xin J Wang D. Clinical and multimodal imaging features of adult-onset neuronal intranuclear inclusion disease. Neurol Sci. (2024) 45:5795–805. doi: 10.1007/s10072-024-07699-y, 39023713 PMC11554744

[13] Zeng T Chen Y Huang H Li S Huang J Xie H . Neuronal intranuclear inclusion disease with NOTCH2NLC GGC repeat expansion: a systematic review and challenges of phenotypic characterization. Aging Dis. (2024) 16:578. doi: 10.14336/AD.2024.0131-1 38377026 PMC11745434

[14] Wu C Wang M Wang X Li W Li S Chen B . The genetic and phenotypic spectra of adult genetic leukoencephalopathies in a cohort of 309 patients. Brain. (2023) 146:2364–76. doi: 10.1093/brain/awac426, 36380532 PMC10232248

[15] Tai H Wang A Zhang Y Liu S Pan Y Li K . Clinical features and classification of neuronal Intranuclear inclusion disease. Neurol Genet. (2023) 9:e200057. doi: 10.1212/NXG.0000000000200057, 37090934 PMC10117695

[16] Xie F Hu X Liu P Zhang D. A case report of neuronal intranuclear inclusion disease presenting with recurrent migraine-like attacks and cerebral edema: a mimicker of MELAS. Front Neurol. (2022) 13:837844. doi: 10.3389/fneur.2022.837844, 35299615 PMC8920963

[17] Su N Mao HJ Mao CH Cui LY Zhu YC Zhou Y . Recurrent headache and visual symptoms in a young man: a rare neuronal intranuclear inclusion disease case report. BMC Neurol. (2022) 22:401. doi: 10.1186/s12883-022-02936-3, 36324076 PMC9628060

[18] Qin X Chen H Zhou C Wang X Gao J Guo N . Neuronal intranuclear inclusion disease: two case report and literature review. Neurol Sci. (2021) 42:293–6. doi: 10.1007/s10072-020-04613-0, 32839883

[19] Liu Y Zeng L Yuan Y Wang Y Chen K Chen Y . Case report: two siblings with neuronal intranuclear inclusion disease exhibiting distinct clinicoradiological findings. Front Neurol. (2022) 13:1013213. doi: 10.3389/fneur.2022.1013213, 36388211 PMC9642335

[20] Yokoi S Yasui K Hasegawa Y Niwa K Noguchi Y Tsuzuki T . Pathological background of subcortical hyperintensities on diffusion-weighted images in a case of neuronal intranuclear inclusion disease. Clin Neuropathol. (2016) 35:375–80. doi: 10.5414/NP300961, 27719745

[21] Ishiura H Shibata S Yoshimura J Suzuki Y Qu W Doi K . Noncoding CGG repeat expansions in neuronal intranuclear inclusion disease, oculopharyngodistal myopathy and an overlapping disease. Nat Genet. (2019) 51:1222–32. doi: 10.1038/s41588-019-0458-z, 31332380

[22] Deng J Gu M Miao Y Yao S Zhu M Fang P . Long-read sequencing identified repeat expansions in the 5'UTR of the NOTCH2NLC gene from Chinese patients with neuronal intranuclear inclusion disease. J Med Genet. (2019) 56:758–64. doi: 10.1136/jmedgenet-2019-106268, 31413119

[23] Lu X Hong D. Neuronal intranuclear inclusion disease: recognition and update. J Neural Transm. (2021) 128:295–303. doi: 10.1007/s00702-021-02313-3, 33599827

[24] Wang Y Wang B Wang L Yao S Zhao J Zhong S . Diagnostic indicators for adult-onset neuronal intranuclear inclusion disease. Clin Neuropathol. (2020) 39:7–18. doi: 10.5414/NP301203, 31661069

[25] Sugiyama A Sato N Kimura Y Maekawa T Enokizono M Saito Y . MR imaging features of the cerebellum in adult-onset neuronal intranuclear inclusion disease: 8 cases. AJNR Am J Neuroradiol. (2017) 38:2100–4. doi: 10.3174/ajnr.A5336, 28818825 PMC7963582

[26] Sone J Ueno S Akagi A Miyahara H Tamai C Riku Y . NOTCH2NLC GGC repeat expansion causes retinal pathology with intranuclear inclusions throughout the retina and causes visual impairment. Acta Neuropathol Commun. (2023) 11:71. doi: 10.1186/s40478-023-01564-3, 37131242 PMC10152767

[27] Jiao B Zhou L Zhou Y Weng L Liao X Tian Y . Identification of expanded repeats in NOTCH2NLC in neurodegenerative dementias. Neurobiol Aging. (2020) 89:142.e1–7. doi: 10.1016/j.neurobiolaging.2020.01.010, 32081467

[28] Bao L Zuo D Li Q Chen H Cui G. Current advances in neuronal intranuclear inclusion disease. Neurol Sci. (2023) 44:1881–9. doi: 10.1007/s10072-023-06677-0, 36795299

[29] Carpenter NJ. Genetic anticipation. Expanding tandem repeats. Neurol Clin. (1994) 12:683–97. doi: 10.1016/S0733-8619(18)30071-9, 7845337

